# Pneumocephalus as result of nonsurgical peri‐implantitis treatment with an air‐polishing device for submucosal debridement—A case report

**DOI:** 10.1002/cre2.578

**Published:** 2022-05-03

**Authors:** Corinna Bruckmann, Lukas Bruckmann, André Gahleitner, Andreas Stavropoulos, Kristina Bertl

**Affiliations:** ^1^ Division of Conservative Dentistry and Periodontology, University Clinic of Dentistry Medical University of Vienna Austria; ^2^ Neurosurgical Department Klinik Landstrasse Vienna Austria; ^3^ Department of Biomedical Imaging and Image‐Guided Therapy Medical University of Vienna Vienna Austria; ^4^ Department of Periodontology, Faculty of Odontology University of Malmö Malmö Sweden; ^5^ Division of Oral Surgery, University Clinic of Dentistry Medical University of Vienna Vienna Austria

**Keywords:** air‐polishing, emphysema, peri‐implantitis, pneumocephalus

## Abstract

**Background:**

A subcutaneous emphysema is an infrequent but potentially life‐threatening complication after dental treatment involving instruments functioning with pressurized air. Emphysemata after the use of high‐speed handpieces and air‐syringes are well documented, however, more recently several reports on emphysemata produced by air‐polishing devices during management of peri‐implant biological complications have appeared. To the best of our knowledge, direct development of pneumocephalus after a dental procedure has never been reported before. Introduction of air likely contaminated with oral bacteria to the intracranial space bares the risk of developing meningitis.

**Case Presentation:**

This case report describes the spreading of a subcutaneous emphysema into the intracranial space (i.e., development of a pneumocephalus) after treatment of a peri‐implantitis lesion with an air‐polishing device equipped with the nozzle for submucosal debridement. A subcutaneous emphysema was noticed during the use of an air‐polishing device and the subsequent computed tomography (CT) examination revealed a quite unexpected spreading of the emphysema into the intracranial space. The patient was admitted to the hospital for close surveillance, CT follow‐up, and intravenous antibiotics to prevent the development of meningitis due to the introduction of air—likely contaminated with oral bacteria—into the intracranial space. After 3 days, the patient was discharged in good condition without any further complications.

**Conclusion:**

In case of an extensive subcutaneous emphysema as result of a dental procedure, a more extended radiographic examination including the mediastinal and cranial space should be considered, to assess the risk for potentially life‐threatening complications.

## 1 INTRODUCTION

A subcutaneous emphysema is an infrequent but potentially life‐threatening complication after dental treatment. The first case report describing such a complication was published in 1900 (Turnbull, 1900), and regarded a patient who played a bugle soon after tooth extraction and developed an enormous swelling of the face on the side of extraction. Reviews (Busuladzic et al., 2020; Heyman & Babayof, 1995; McKenzie & Rosenberg, 2009) have summarized published cases of subcutaneous emphysemata in relation to dental treatment, summing up to about 150 cases within the last 60 years (i.e., from 1960 to 2018); most of the reported emphysemata were caused by air‐driven handpieces and air syringes. However, as air‐polishing devices are frequently used in the management of peri‐implant biological complications (Klinge et al., 2018), it is not surprising that emphysemata have also been reported after their use (Alonso et al., 2017; Bassetti et al., 2014; La Monaca et al., 2021; Lee et al., 2018).

Emphysemata can usually spread into the facial, (peri‐)orbital, pharyngeal, cervical, and/or mediastinal regions. Rarely, air can be introduced into the circulatory system causing systemic air embolism, as observed in a case of a subcutaneous emphysema spreading to the mediastinum, that caused cerebral hypoperfusion (Magni et al., 2008). In most cases, the emphysema resorbs spontaneously within a few days and complete recovery is achieved after 7–10 days, while a surgical intervention is only seldomly required in cases with (suspected) infection and/or incomplete resolution (Busuladzic et al., 2020; Cardo et al., 1972; Lee et al., 2018; McKenzie, & Rosenberg, 2009). Potentially life‐threatening complications are infections due to the introduction of oral bacteria into the fascial spaces (e.g., necrotizing fasciitis, or mediastinitis) and/or the resulting mechanical compression of surrounding tissues/organs due to the associated internal swelling (e.g., pneumopericardium, tracheal compression) (McKenzie & Rosenberg, 2009; Mascarenhas, 2019). Therefore, in most cases antibiotics are prescribed together with analgesic medication for pain control; the additional prescription of steroids and/or 100% O_2_ supplementation may also be considered. In severe cases, patients are admitted to the hospital for close surveillance and administration of intravenous antibiotics (Busuladzic et al., 2020; Heyman & Babayof, 1995; McKenzie & Rosenberg, 2009; Lee et al., 2018).

The present case report is—to the best of our knowledge—the first to report on the direct introduction of air into the intracranial space (i.e., pneumocephalus) after a dental procedure, specifically, after submucosal debridement of a peri‐implantitis lesion with an air‐polishing device.

## 2 CASE REPORT

The present case report follows the CARE guidelines (Riley et al., 2017) (Supporting Information: Appendix S1) and the patient has provided a signed informed consent on publishing the case including clinical pictures and radiographs.

**Figure 1:**
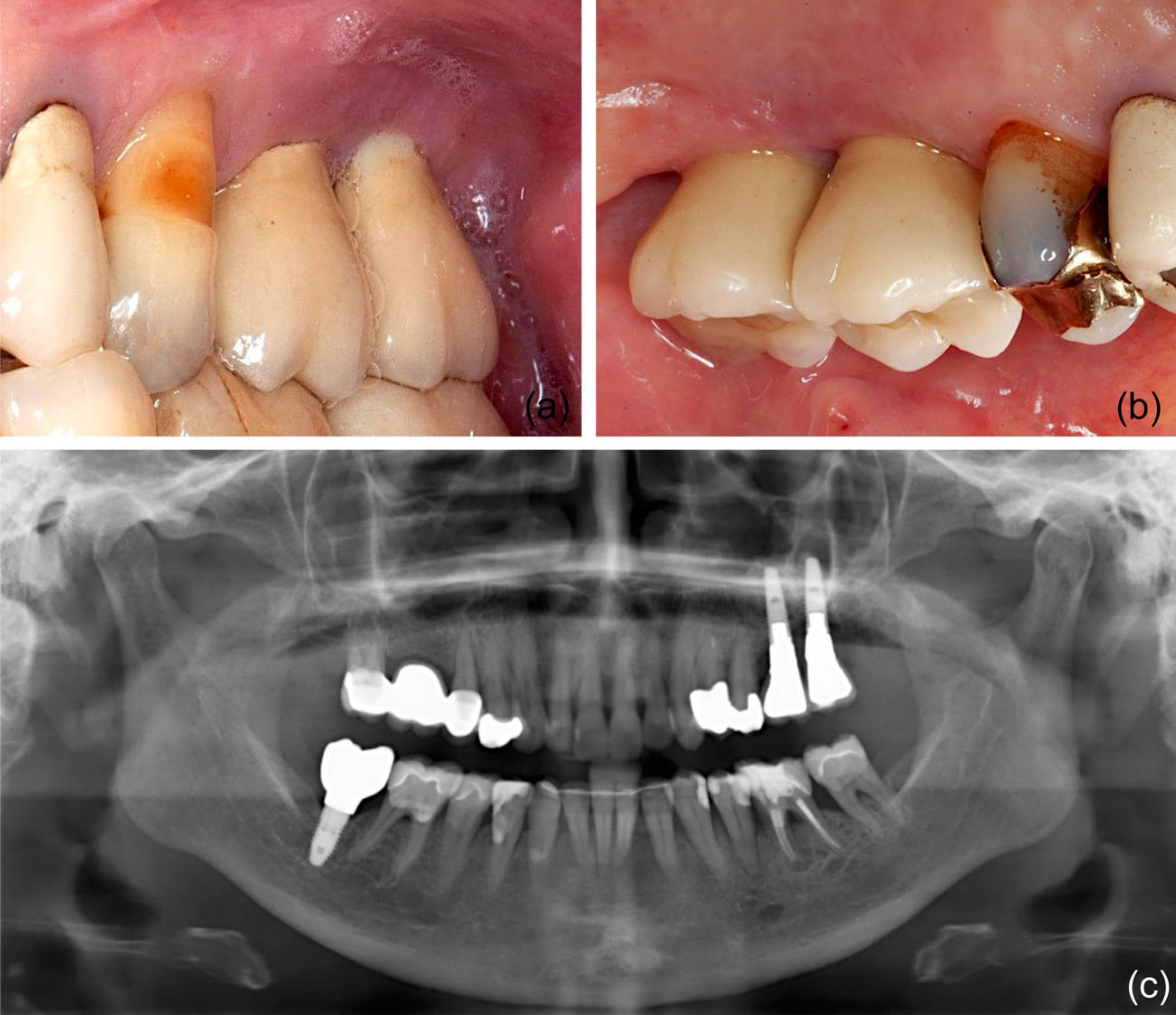
Clinical (buccal [a] and palatal [b]) and radiographic (c) view of the implants at positions 26 and 27. Both implants present a wide band of keratinized mucosa and have been installed after a sinus floor elevation procedure.

**Figure 2:**
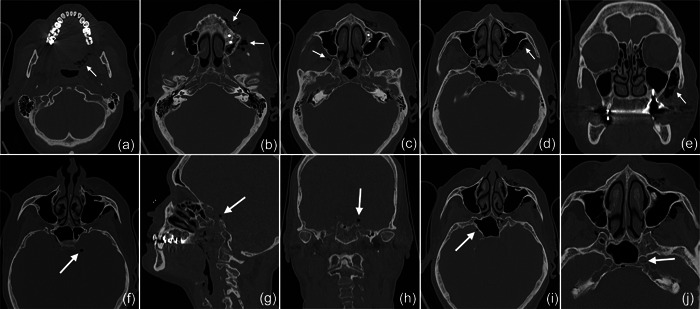
The subcutaneous emphysema extended on the left side pharyngeal (a) and facial (b), into the right (c) and left (d) pterygopalatine fossa with a maximum extension of up to 3 cm on the left side (e). The intracranial air bubbles reached a diameter of up to 5 mm (axial [f], sagittal [g], and coronal [h] view) and were detected on both sides of the sella turcica in the region of the cavernous sinus (i) and on the left side in the carotid canal (j).

**Figure 3:**
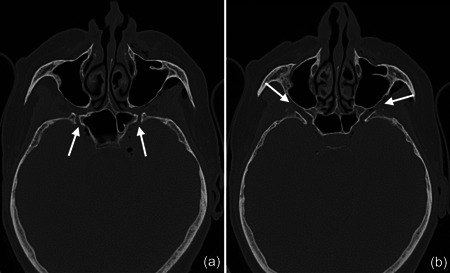
The pterygoid canal (a) and the forum rotundum (b) could be considered as potential entry points for the air into the intracranial space; especially within the canal leading to the foramen rotundum several smaller air bubbles were captured on the CT scan (b). CT, computed tomography.

**Figure 4:**
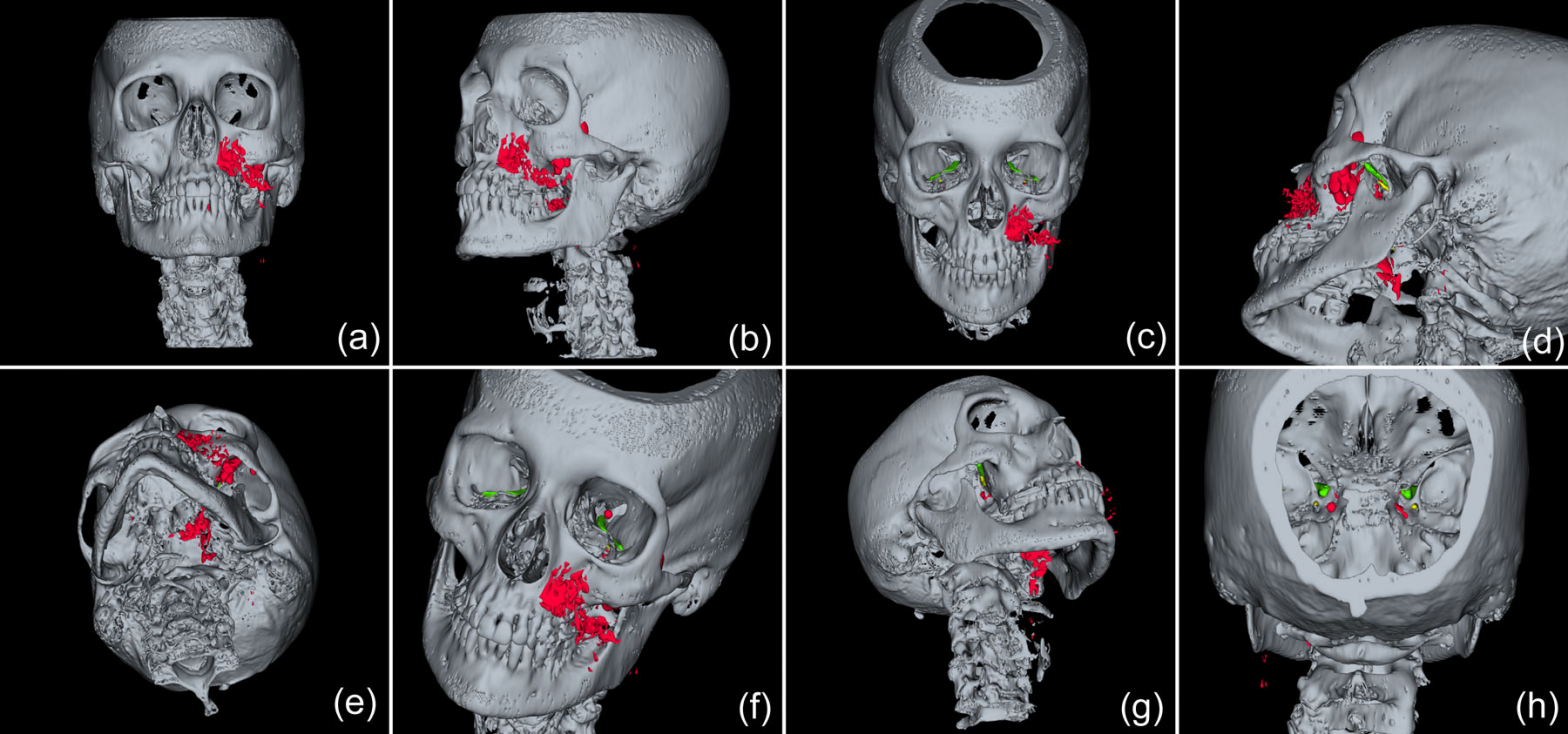
3D reconstruction (a‐h) of the emphysema (red) and display of the potential pathways to the intracranial space, i.e., along the inferior orbital fissure and finally via the foramen rotundum (green) and/or via the pterygoid canal (yellow). 3D, three‐dimensional.

In 2019, a female patient, 62 years old, without any current remarkable systemic health issues, was enrolled in a routine supportive periodontal and peri‐implant care program at the Department of Conservative Dentistry and Periodontology (University Clinic of Dentistry, Medical University of Vienna, Austria) for treatment of peri‐implantitis. The patient had received, in 2012, a sinus floor elevation in the region of 26 and 27, with the lateral window technique and the use of deproteinized bovine bone mineral and a collagen membrane (BioOss® and BioGide®; Geistlich Pharma AG, Wolhulsen, CH). No perforation of the sinus membrane was reported and, postoperative healing was uneventful. Four months later, two implants were installed (Replace Select Tapered; Nobel Biocare™ Services AG, Zürich, CH). All surgical procedures were performed at the Department of Oral Surgery (University Clinic of Dentistry, Medical University of Vienna, Austria), while the prosthetic treatment and peri‐implant maintenance care were carried out elsewhere. In 2019, the patient presented with probing pocket depths (PD) of 6–7 mm at the implants in regions 26 and 27, including bleeding on probing and pus discharge. Following site‐specific reinforcement of oral hygiene with adjustment of interdental cleaning devices, and successful smoking cessation intervention, the patient received two rounds of nonsurgical peri‐implantitis therapy with handsonic and ultrasonic instruments and adjunct systemic and local antibiotics, respectively, during 2019–2020. When the patient returned in the clinic in March 2021, after the covid‐19 disruptions, PD was again 7 mm at the implant 27 (Figure 1). For the treatment at this time the choice was made to use an air‐polishing device (Airflow® One; E.M.S. Electro Medical Systems S.A., Nyon, CH). The supragingival handpiece (Airflow® handpiece) was used with an Erythritol‐based powder (Airflow® Powder PLUS; E.M.S. Electro Medical Systems S.A., Nyon, CH) for the removal of supragingival and subgingival biofilm according to the manufacturer's recommendations, that is, the handpiece was positioned at a 60° angle in a distance of 3–5 mm towards the gingival sulcus. The settings were at 100% water flow and 40% power. For the treatment of the 7 mm pocket at the mesial aspect of implant 27, the subgingival handpiece (Perioflow® handpiece) with a sterile disposable nozzle was used with the same Erythritol‐based powder and settings (100% water flow and 40% power). After inserting the nozzle mesially up to the previously measured depth of 7 mm, the device was activated. Almost immediately, that is, after 2 s, the patient uttered extreme discomfort in the left side of her face and head, upon which the treatment was stopped, and the patient brought into an upright position. An emergency examination showed an awake and orientated patient, who reported minimal shortness of breath and pain behind the left eye, as well as slight dysphagia on the left side of the throat. Light reaction of pupils, heart rate (72 per minute), and blood pressure (135/85 mmHg) were normal. Quick neurologic examination of the cranial nerve function was inconspicuous. Extraoral and intraoral inspection of the soft tissues showed no erythema or other pathologic findings; palpation of the cheeks and the retromandibular and submandibular areas gave no hint for swelling or crepitus. Nevertheless, due to the strange sensations reported by the patient and unclear clinical signs, a low‐dose dental computed tomography (CT) scan was recorded (axial, 120 kV, 140 mAs, 512 matrix, 0.5 mm thickness, 0.5 mm increment, native, high‐resolution bone window; Somatom Definition AS, Siemens Healthcare, Erlangen, GER). The CT scan confirmed the development of a subcutaneous emphysema. The air spread from the facial aspect of the posterior left maxilla on the same side into a caudal (i.e., pharyngeal) and on both sides into a cranial (i.e., into the left and right pterygopalatine fossa) direction (Figure 2a–e); on the left side the emphysema reached a dimension of up to 3 cm in the pterygopalatine fossa (Figure 2e). Further, air accumulation was also detected in the left carotid canal (Figure 2j), in the canal leading to the foramen rotundum (Figure 3b), and intracranially on both sides of the sella turcica in the region of the cavernous sinus, with a maximum diameter of up to 5 mm (Figure 2f–i). Three‐dimensional reconstruction of the emphysema is presented in Figure 4.

**Figure 5:**
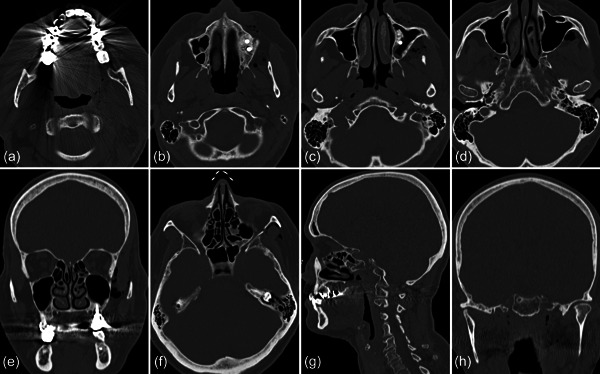
A CT scan on the next day indicated resolution of the intracranial air and regression of the subcutaneous emphysema. The section plane and level in a‐h correspond to the section plane and level presented in a‐h of Figure 2. CT, computed tomography.

Because of this quite unexpected and untypical finding of intracranial air and due to the risk for meningitis from the likely introduction of oral bacteria together with the air, the patient was referred to the Emergency Department of the adjacent Vienna General Hospital. The patient was kept under surveillance as an inpatient and received intravenous antibiotic treatment (ceftriaxone 2 g once per day and ampicilline/sulbactame 3 g three times per day). A CT scan on the next day indicated already resolution of the intracranial air and regression of the soft tissue emphysema (Figure 5). The patient was discharged in good condition on Day 3, with prescription of oral antibiotics for another 5 days (amoxicillin 825 mg and clavulanic acid 125 mg twice per day).

Three months later, the clinical situation at the implant 27 had considerably worsened with a PD of 12 mm and the patient agreed to have the implant removed. Explantation and postoperative healing were uneventful, without any oro‐antral communication.

## 3 DISCUSSION

Since 1960, about 150 case reports have been published on the development of a subcutaneous emphysema in relation to a dental procedure (Busuladzic et al., 2020; Heyman & Babayof, 1995; McKenzie & Rosenberg, 2009). These reports described emphysemata in quite diverse locations, spreading from the orbital region to the mediastinum. In the present case, we observed introduction of air into the intracranial space; to the best of our knowledge, direct development of pneumocephalus resulting from a dental procedure has not been reported before.

According to a review (Markham, 1967), summarizing 295 cases with pneumocephalus, the cause for intracranial air accumulation is most often trauma leading to fractures of the skull and especially of the facial skeleton, for example after automobile or motorcycle accidents. Pneumocephalus is also frequently related to neurosurgical interventions, neoplasm, and infections involving the skull (Markham, 1967; Schirmer et al., 2010). Rarely, pneumocephalus may also develop in the absence of any fracture and/or visible lacerations on the skin or conjunctiva after compressed‐air injury to the eye (Akbari‐Kamrani et al., 2020; Bagheri et al., 2018; Hiraoka et al., 2013; Koenig, 1977; Lubniewski & Feibel, 1989; Williams & Frankel, 1999; Yuksel et al., 2007). It is assumed, that compressed air may enter the retrobulbar space via some invisible disruption and micro‐lacerations of the conjunctiva or that it may even be able to pass through intact conjunctival tissue (Hiraoka et al., 2013), and that the air enters then the intracranial space via the optic canal (Williams & Frankel, 1999; Yuksel et al., 2007) and/or superior orbital fissure (Lubniewski & Feibel, 1989).

**Figure 6:**
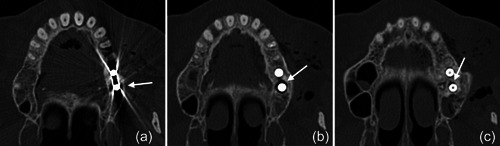
The implant in position 27 presented with a sufficient buccal bone wall (a), but the peri‐implant bone defect (b) extended at the mesial aspect far apically (c) potentially in connection via the augmentation material with the residual bone defect in the lateral sinus wall presented in Figure 7.

**Figure 7:**
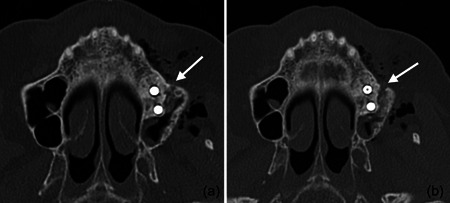
A residual bone defect in the lateral sinus wall from the previous lateral window technique (a, b) appears to be approximately at the level of the apex of the implant in position 27. The compressed air might have extended along the peri‐implant defect (Figure 6) into the augmentation material and exit into the surrounding soft tissue of the facial aspect of the posterior left maxilla via this residual bone defect in the lateral sinus wall.

In the present case, where a pneumocephalus developed during submucosal debridement of a peri‐implantitis lesion with an air‐polishing device, the following pathway for the intracranial entrance of the air is suspected based on the localization of air, registered in the diagnostic CT taken after the procedure. Based on this CT scan, and although it is acknowledged that the detection of a thin buccal bone wall in CT/CBCT scans is largely inaccurate (Domic et al., 2021), it appears that the implant in position 27 had an intact buccal bone at its marginal aspect (Figure 6a). However, it had a very deep peri‐implant bone defect at its mesial aspect, extending almost to the apex of the implant, that is, into the augmented sinus space (Figure 6b,c). Additionally, there was a bone defect at the buccal aspect of the sinus wall—most likely a remnant of the lateral window made during the sinus floor elevation procedure—at a similar level as the bottom of the intrabony defect (Figure 7). Hence, as the nozzle was inserted at the mesial aspect of the implant, the compressed air could have extended along the peri‐implant defect into the augmentation material and then exited via this residual bone defect in the lateral sinus wall into the surrounding soft tissue of the facial aspect of the posterior left maxilla. From there, the air could have spread on the same side into a caudal (i.e., pharyngeal) and on both sides into a cranial (i.e., into the left and right pterygopalatine fossa) direction (Figure 2). Further, it appeared that it progressed along the inferior orbital fissure and finally entered the intracranial space via the foramen rotundum and/or the pterygoid canal (i.e., vidian canal) (Figures 2, 3, 4).

A patient diagnosed with pneumocephalus should be immediately admitted to a hospital. Keeping the patient as an inpatient allows close surveillance, including bed rest, and the possibility for appropriate treatment. This can include intravenous administration of antibiotics to prevent the development of meningitis and of antipyretic pain medication to prevent hyperthermia, supplementation with 100% O_2_ for a faster resorption rate, and/or serial CT scans to monitor gradual resorption of the intracranial air (Gorissen et al., 2019; Schirmer et al., 2010). It should be noted that antibiotic prophylaxis in case of pneumocephalus to prevent meningitis is controversially discussed, for example, antibiotic prophylaxis is not supported by the literature for patients with basilar skull fractures (Nellis et al., 2014; Ratilal et al., 2015). Nevertheless, the likely introduction of oral bacteria into the intracranial space was regarded as additional risk factor for meningitis, and therefore the current patient received intravenous antibiotics at the admitting hospital.

In previous publications reporting on emphysemata by means of an air‐polishing device, during the nonsurgical treatment of peri‐implantitis, the lack of a buccal bone wall and/or keratinized buccal mucosa have been discussed as potential risk factors for their development (Alonso et al., 2017; Bassetti et al., 2014; Lee et al., 2018), but this was not the case for the current patient (Figures 1 and 6a). Another relevant parameter seems to be the air‐pressure exiting from the various tips, of the various handpieces, at various power settings, but there is only limited information available on this parameter among the various devices in the market. For example, according to the manufacturer of the device used in this report, the exiting pressure from the disposable nozzle of the subgingival handpiece—within a periodontal/peri‐implant pocket—can reach maximum 1.8 bar. Further, treatment time may potentially affect the risk of emphysema development. It seems reasonable to assume that with longer treatment time, close to the bottom of the defect, and at a given power setting, the risk for emphysema development may be higher as larger amounts of pressurized air would be delivered and the tissues might become less resilient. However, shorter treatment time might result in a less clean implant surface compared to longer treatment time (Bertl et al., 2021). In perspective, the maximum pressure and/or time of treatment below which no emphysema may occur is currently unknown and probably impossible to estimate. It seems reasonable that the likelihood to develop a subcutaneous emphysema depends on the interplay among local anatomical conditions (e.g., defect extent, tissue consistency), hardware specifications (e.g., exiting pressure, tip‐design), and the way of using the device (e.g., appropriate setting adjustment to the specific site, duration of use). Considering the facts that the number of implants placed is increasing and that peri‐implant biological complications are frequent, as well as that air‐polishing devices are often used in their management (Klinge et al., 2018), it is reasonable to expect that emphysemata may become more frequent. Thus, further research is needed to provide evidence‐based recommendations for the safe use of air‐polishing devices in the management of peri‐implantitis.

## 4 CONCLUSION

The present case of direct development of pneumocephalus from a dental procedure is the first in the literature, and thus it should be regarded as an extremely rare complication. However, it is suggested that in case of an extensive subcutaneous emphysema, a more extended radiographic examination including the cranial and mediastinal space should be considered as standard examination, to assess the risk of potential life‐threatening complications.

## AUTHOR CONTRIBUTIONS

*Conceptualization and manuscript drafting and reviewing*: Corinna Bruckmann, Kristina Bertl, Andreas Stavropoulos. *Image interpretation and manuscript drafting and reviewing*: André Gahleitner, Lukas Bruckmann.

## CONFLICTS OF INTEREST

The authors declare no conflicts of interest.

## Supporting information

Supporting information.

## Data Availability

Data available by the authors upon reasonable request.

## References

[cre2578-bib-0001] Akbari‐Kamrani, M. , Akbari‐Kamrani, B. , & Tavakoli, M. (2020). Urgent subconjunctival needle decompression for orbital compartment emphysema caused by compressed air injury. Annals of Emergency Medicine, 76, 801–803. 10.1016/j.annemergmed.2020.06.025 32950279

[cre2578-bib-0002] Alonso, V. , García‐Caballero, L. , Couto, I. , Diniz, M. , Diz, P. , & Limeres, J. (2017). Subcutaneous emphysema related to air‐powder tooth polishing: A report of three cases. Australian Dental Journal, 62, 510–515. 10.1111/adj.12537 28590506

[cre2578-bib-0003] Bagheri, A. , Veisi, A. , Memarzade, S. E. , & Tavakoli, M. (2018). Orbital, periorbital, and intracranial emphysema caused by compressed air injury in a 5‐year‐old child. Ophthalmic Plastic and Reconstructive Surgery, 34, e151–e153. 10.1097/IOP.0000000000001165 29952933

[cre2578-bib-0004] Bassetti, M. , Bassetti, R. , Sculean, A. , & Salvi, G. E. (2014). [Subcutaneous emphysema following non‐surgical peri‐implantitis therapy using an air abrasive device: A case report]. Swiss Dental Journal, 124, 807–817. 25118639 10.61872/sdj-2014-07-08-04

[cre2578-bib-0005] Bertl, K. , Isik, A. , Truong, T. , Heimel, P. , & Stavropoulos, A. (2021). Efficacy of air‐polishing devices in disinfecting implant surfaces in a non‐surgical simulation. Clinical Oral Implants Research, 32, S22. 10.1111/clr.14452 PMC12423577 40531059

[cre2578-bib-0006] Busuladzic, A. , Patry, M. , Fradet, L. , Turgeon, V. , & Bussieres, M. (2020). Cervicofacial and mediastinal emphysema following minor dental procedure: A case report and review of the literature. Journal of Otolaryngology‐Head & Neck Surgery, 49, 61. 10.1186/s40463-020-00455-0 32811562 PMC7433085

[cre2578-bib-0007] Cardo, V. A. , Mooney, J. W. , & Stratigos, G. T. (1972). Iatrogenic dental‐air emphysema: Report of case. Journal of the American Dental Association, 85, 144–147. 10.14219/jada.archive.1972.0283 4555336

[cre2578-bib-0008] Domic, D. , Bertl, K. , Ahmad, S. , Schropp, L. , Hellén‐Halme, K. , & Stavropoulos, A. (2021). Accuracy of cone‐beam computed tomography is limited at implant sites with a thin buccal bone: A laboratory study. Journal of Periodontology, 92, 592–601. 10.1002/JPER.20-0222 32846005 PMC8247288

[cre2578-bib-0009] Gorissen, Z. , Hakvoort, K. , van den Boogaart, M. , Klinkenberg, S. , & Schijns, O. (2019). Pneumocephalus: A rare and life‐threatening, but reversible, complication after penetrating lumbar injury. Acta Neurochirurgica (Wien), 161, 361–365. 10.1007/s00701-018-03796-y PMC6373275 30652201

[cre2578-bib-0010] Heyman, S. N. , & Babayof, I. (1995). Emphysematous complications in dentistry, 1960‐1993: An illustrative case and review of the literature. Quintessence International, 26, 535–543. 8602428

[cre2578-bib-0011] Hiraoka, T. , Ogami, T. , Okamoto, F. , & Oshika, T. (2013). Compressed air blast injury with palpebral, orbital, facial, cervical, and mediastinal emphysema through an eyelid laceration: A case report and review of literature. BMC Ophthalmology, 13, 68. 10.1186/1471-2415-13-68 24195485 PMC4228247

[cre2578-bib-0012] Klinge, B. , Klinge, A. , Bertl, K. , & Stavropoulos, A. (2018). Peri‐implant diseases. European Journal of Oral Sciences, 126(Suppl 1), 88–94. 10.1111/eos.12529 30178555

[cre2578-bib-0013] Koenig, R. P. (1977). Traumatic eye and intracranial air‐movement from a subconjunctival to an intracranial position. American Journal of Ophthalmology, 83, 915–917. 10.1016/0002-9394(77)90924-2 868996

[cre2578-bib-0014] Lee, S. T. , Subu, M. G. , & Kwon, T. G. (2018). Emphysema following air‐powder abrasive treatment for peri‐implantitis. Maxillofacial Plastic and Reconstructive Surgery, 40, 12. 10.1186/s40902-018-0151-7 29774206 PMC5949097

[cre2578-bib-0015] Lubniewski, A. J. , & Feibel, R. M. (1989). Traumatic air blast injury with intracranial, bilateral orbital, and mediastinal air. Ophthalmic Surgery, 20, 677–679. 2812697

[cre2578-bib-0016] Magni, G. , Imperiale, C. , Rosa, G. , & Favaro, R. (2008). Nonfatal cerebral air embolism after dental surgery. Anesthesia and Analgesia, 106, 249–251. 10.1213/01.ane.0000289634.24785.04 18165585

[cre2578-bib-0017] Markham, J. W. (1967). The clinical features of pneumocephalus based upon a survey of 284 cases with report of 11 additional cases. Acta Neurochirurgica (Wien), 16, 1–78. 10.1007/BF01401900 6032371

[cre2578-bib-0018] Mascarenhas, R. J. (2019). Management of subcutaneous facial emphysema secondary to a class V dental restoration. Clinical Case Reports, 7, 1025–1030. 10.1002/ccr3.2141 31110739 PMC6509900

[cre2578-bib-0019] McKenzie, W. S. , & Rosenberg, M. (2009). Iatrogenic subcutaneous emphysema of dental and surgical origin: A literature review. Journal of Oral and Maxillofacial Surgery, 67, 1265–1268. 10.1016/j.joms.2008.12.050 19446214

[cre2578-bib-0020] La Monaca, G. , Pranno, N. , Annibali, S. , Vozza, I. , & Cristalli, M. P. (2021). Subcutaneous facial emphysema following open‐flap air‐powder abrasive debridement for peri‐implantitis: A case report and an overview. International Journal of Environmental Research and Public Health, 18, 13286. 10.3390/ijerph182413286 34948898 PMC8702083

[cre2578-bib-0021] Nellis, J. C. , Kesser, B. W. , & Park, S. S. (2014). What is the efficacy of prophylactic antibiotics in basilar skull fractures. Laryngoscope, 124, 8–9. 10.1002/lary.23934 24122671

[cre2578-bib-0022] Ratilal, B. O. , Costa, J. , Pappamikail, L. , & Sampaio, C. (2015). Antibiotic prophylaxis for preventing meningitis in patients with basilar skull fractures. Cochrane Database of Systematic Reviews, 4, CD004884. 10.1002/14651858.CD004884.pub4 PMC10554555 25918919

[cre2578-bib-0023] Riley, D. S. , Barber, M. S. , Kienle, G. S. , Aronson, J. K. , von Schoen‐Angerer, T. , Tugwell, P. , Kiene, H. , Helfand, M. , Altman, D. G. , Sox, H. , Werthmann, P. G. , Moher, D. , Rison, R. A. , Shamseer, L. , Koch, C. A. , Sun, G. H. , Hanaway, P. , Sudak, N. L. , Kaszkin‐Bettag, M. , … Gagnier, J. J. (2017). CARE guidelines for case reports: Explanation and elaboration document. Journal of Clinical Epidemiology, 89, 218–235. 10.1016/j.jclinepi.2017.04.026 28529185

[cre2578-bib-0024] Schirmer, C. M. , Heilman, C. B. , & Bhardwaj, A. (2010). Pneumocephalus: Case illustrations and review. Neurocritical Care, 13, 152–158. 10.1007/s12028-010-9363-0 20405340

[cre2578-bib-0025] Turnbull, A. (1900). A remarkable coincidence in dental surgery. British Medical Journal, 1, 1131.

[cre2578-bib-0026] Williams, T. R. , & Frankel, N. (1999). Intracerebral air caused by conjunctival laceration with air hose. Archives of Ophthalmology, 117, 1090–1091. 10448760

[cre2578-bib-0027] Yuksel, M. , Yuksel, K. Z. , Ozdemir, G. , & Ugur, T. (2007). Bilateral orbital emphysema and pneumocephalus as a result of accidental compressed air exposure. Emergency Radiology, 13, 195–198. 10.1007/s10140-006-0546-0 17115095

