# Supplementary material for: Pneumocephalus as result of nonsurgical peri‐implantitis treatment with an air‐polishing device for submucosal debridement—A case report
Source: Clin Exp Dent Res. 2022 May 3;8(3):632–9. doi: 10.1002/cre2.578 (PMC9209792; doi:10.1002/cre2.578)
Supplement: Supplementary file 1 — Supporting information. [file CRE2-8-632-s001.pdf]

CARE Checklist of information to include when writing a case report

CARE case report guidelines

(cc) BY-NC-ND

Topic	Item	Checklist item description	Reported on Line
Title	1	The diagnosis or intervention of primary focus followed by the words "case report"	page 1 line 1-2
Key Words	2	2 to 5 key words that identify diagnoses or interventions in this case report, including "case report"	page 2 line 31
Abstract (no references)	3a	Introduction: What is unique about this case and what does it add to the scientific literature?	page 2 line 2-9
	3b	Main symptoms and/or important clinical findings	page 2 line 7-11
	3c	The main diagnoses, therapeutic interventions, and outcomes	page 2 line 11-14
	3d	Conclusion—What is the main "take-away" lesson(s) from this case?	page 2 line 15-17
Introduction	4	One or two paragraphs summarizing why this case is unique (**may include references**)	page 3 line 2-32
Patient Information	5a	De-identified patient specific information.	page 4 line 6-9
	5b	Primary concerns and symptoms of the patient.	page 4 line 31-35, page 5 line 1-15
	5c	Medical, family, and psycho-social history including relevant genetic information	page 4 line 6-9
	5d	Relevant past interventions with outcomes	page 4 line 9-31
Clinical Findings	6	Describe significant physical examination (PE) and important clinical findings.	page 4 line 31-35, page 5 line 1-24
Timeline	7	Historical and current information from this episode of care organized as a timeline	page 4 line 6-35, page 5 line 1-28
Diagnostic Assessment	8a	Diagnostic testing (such as PE, laboratory testing, imaging, surveys).	page 4 line 34-35, page 5 line 1-24
	8b	Diagnostic challenges (such as access to testing, financial, or cultural)	not relevant
	8c	Diagnosis (including other diagnoses considered)	page 4 line 34-35, page 5 line 1-24
	8d	Prognosis (such as staging in oncology) where applicable	not relevant
Therapeutic Intervention	9a	Types of therapeutic intervention (such as pharmacologic, surgical, preventive, self-care)	page 5 line 17-24
	9b	Administration of therapeutic intervention (such as dosage, strength, duration)	page 5 line 17-24
	9c	Changes in therapeutic intervention (with rationale)	page 5 line 17-24
Follow-up and Outcomes	10a	Clinician and patient-assessed outcomes (if available)	page 5 line 26-28
	10b	Important follow-up diagnostic and other test results	page 5 line 17-28
	10c	Intervention adherence and tolerability (How was this assessed?)	page 5 line 17-28
	10d	Adverse and unanticipated events	page 5 line 26-28
Discussion	11a	A scientific discussion of the strengths AND limitations associated with this case report	page 4 line 31 until page 8 line 2
	11b	Discussion of the relevant medical literature **with references**.	page 4 line 31 until page 8 line 2
	11c	The scientific rationale for any conclusions (including assessment of possible causes)	page 4 line 31 until page 8 line 2
	11d	The primary "take-away" lessons of this case report (without references) in a one paragraph conclusion	page 8 line 5-9
Patient Perspective	12	The patient should share their perspective in one to two paragraphs on the treatment(s) they received	not included
Informed Consent	13	Did the patient give informed consent? Please provide if requested	Yes ☒ No ☐
